# The retrosplenial cortex and long-term spatial memory: from the cell to the network

**DOI:** 10.1016/j.cobeha.2020.01.014

**Published:** 2020-04

**Authors:** Michal M Milczarek, Seralynne D Vann

**Affiliations:** School of Psychology, Cardiff University, Cardiff, UK

## Abstract

In this review we briefly outline how lesion studies, temporary inactivation and neural activity assays have helped update functional models of the retrosplenial cortex, a region critical for episodic and spatial memory. We advocate for the continued importance of appropriately designed behavioural studies in the context of novel experimental methods, such as optogenetic and chemogenetic manipulations. At the same time, we caution against the overreliance on any given level of analysis or experimental technique. Complementary, multimodal strategies are required for understanding how the retrosplenial cortex contributes to the formation and storage of memories both at a structural and systems-level.

## Highlights

• The role of the retrosplenial cortex remains poorly understood in humans.
• Animal models, including rodents, offer improved experimental access and control.
• Combining multimodal approaches supports retrosplenial importance for spatial memory.
• Immediate-early gene imaging can visualise retrosplenial neural ensembles.
• Novel neuroscience techniques must be combined with well-designed behavioural assays.

**Current Opinion in Behavioral Sciences** 2020, **32**:50–56

This review comes from a themed issue on **Understanding memory: Which level of analysis?**

Edited by **Morgan Barense** and **Hugo J Spiers**

For a complete overview see the Issue and the Editorial

Available online 5th March 2020

**https://doi.org/10.1016/j.cobeha.2020.01.014**

2352-1546/© 2020 The Author(s). Published by Elsevier Ltd. This is an open access article under the CC BY license (http://creativecommons.org/licenses/by/4.0/).

## Introduction

**Figure 1:**
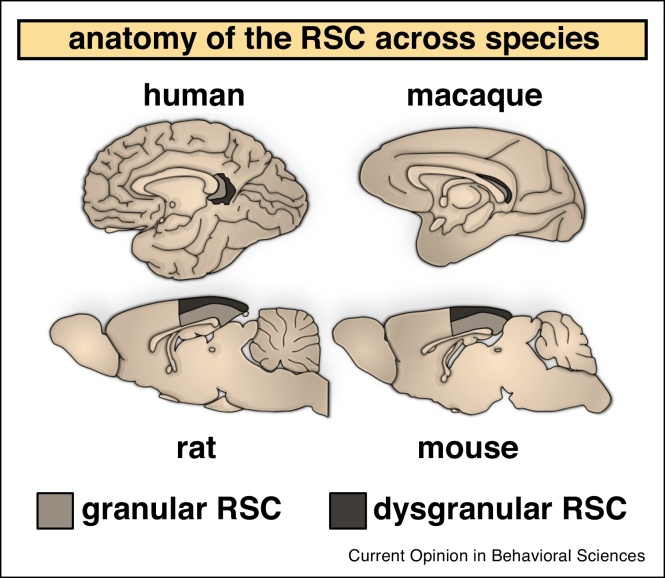
The gross anatomy of the retrosplenial cortex (RSC) across species is preserved. Human and macaque RSC wraps around the caudal aspect of the splenium of the corpus callosum while in rodents, it is located dorsally and spans nearly half the length of the cerebrum. The RSC is subdivided into granular and dysgranular regions, corresponding to Brodmann areas 29 and 30, respectively. Despite differences in relative location and size, primate and rodent RSC displays similar cytoarchitectonic properties and shares homologous connectivity with other regions.

Understanding the physiological basis of memory has been a key goal of neuroscientific research. Much progress has been made by appreciating the multi-level nature of mnemonic processes, from synapses to neurons, within both local and long-range circuits that extend across networks. However, memory research has placed an overwhelming focus on the role and properties of a single brain site, the hippocampal formation, downplaying essential contributions from other areas. Among them, the retrosplenial cortex (RSC; Brodmann areas 29&30) has long been implicated in memory — principally due to its connectivity to other memory-related areas [1] — but the last twenty years has seen its role in memory and navigation firmly cemented. Increased interest in RSC arose in the main part from functional magnetic resonance imaging (fMRI) studies that repeatedly implicated the posterior cingulate/RSC in memory, navigation, scene processing and the default mode network [2]. Despite these advances, research in humans can prove difficult due to the location of the RSC — situated deep in the callosal sulcus (see Figure 1) — and difficulty in dissociating RSC involvement from that of adjacent regions [2]. By contrast, the relative size and accessibility of the rodent RSC (see Figure 1), combined with similarities in structure and connectivity across species [2], reinforces the usefulness of rodent models for examining RSC contributions. Rodent studies have enabled us to develop mechanistic models of RSC function, thus complementing the correlative analyses carried out using fMRI studies in humans. Over the following sections, we will discuss how multi-level and cross-species approaches have proved particularly informative for studying the RSC, and memory in general.

## RSC lesions studies: identifying the necessity of RSC for mnemonic processes

Neuropsychological studies involving patients with damage to specific brain areas have been the bedrock of memory research. There are remarkably few reports of patients with RSC lesions but, from the studies that are available, patients typically present with spatial disorientation and anterograde amnesia, which is sometimes accompanied by retrograde and semantic memory impairments [2,3]. However, it is difficult to ascribe these functions specifically to RSC given that lesions are rarely circumscribed. In contrast, targeted lesions in rodents have confirmed the role of RSC in both spatial and non-spatial memory [2,4]. Studies in animals have also shown RSC-lesion effects are often most evident when animals are required to switch between frames of reference, that is, egocentric or allocentric viewpoints, or integrate information across different sensory modalities [5,6].

Rodent studies can be particularly informative as they enable specific subregions within RSC to be targeted; for example, lesions studies have examined the contributions of granular and dysgranular subdivisions and identified a general role for granular RSC in spatial memory but a more focused role for dysgranular RSC in visuo-spatial processing [7,8] (Figure 1). Furthermore, impairments following selective lesions along the rostro-caudal axis of RSC are consistent with a distribution of function along the length of RSC [9,10]. Together, lesion studies in rodents have advanced our understanding of the RSC and highlighted the heterogeneity within the RSC, which would be missed by simply focusing on the RSC as a single structure.

Lesion studies have typically assessed the effects of RSC damage on new learning, that is, lesions are made before behavioural training. From patient studies, RSC pathology results in retrograde amnesia for autobiographical episodes and impairs the use of previously learnt spatial information [3,11], highlighting the need to assess post-training lesions in rodents. Findings from the few studies available are consistent with RSC being important for the long-term storage or retrieval of previously learnt information. Todd *et al.* [12] showed that retrieval of auditory fear memories was disrupted when RSC lesions were made several weeks after encoding. Likewise, post-training RSC lesions impaired rats’ ability to discriminate between previously rewarded arms of a radial-arm maze (RAM), irrespective of whether the training occurred 4-weeks or one day before surgery [13]. Importantly, these retrograde memory impairments appear consistent across species: Buckley and Mitchell showed that RSC lesions in macaques disrupted performance on an object-scene memory task that had been learnt before surgery [14].

## Limitations of lesions: a move to temporary inactivation

Traditional lesion studies have provided a wealth of information regarding the role of RSC for memory but there are limitations to this approach. Compensatory mechanisms can minimise the impact of the lesions, particularly for tasks requiring slow acquisition. This is especially pertinent for the RSC as it may explain why deficits following RSC lesions are often mild and/or resolve with continued training. This may account for the apparent mismatch between the prevalence of RSC involvement in human fMRI studies and the somewhat mild effects of RSC lesions in rodents.

Temporary inactivation enables the RSC to be silenced at different stages of learning, which reduces the likelihood of animals using compensatory strategies. In rodents, only a couple of studies have used drug infusions to temporarily inactivate the RSC during spatial tasks [e.g. 15]. While impairments of T-maze alternation following RSC lesions are only observed when intra-maze and extra-maze cues are put in conflict, rats infused with muscimol into the RSC show deficits even on the standard version of the task [16]. The importance of RSC for fear memory consolidation has been shown using temporary inactivation with infusates such as muscimol as well as with compounds that interfere with post-learning protein synthesis or immediate-early gene (IEG) expression [12,17, 18, 19]. The move to chemogenetic and optogenetic approaches [4] has the potential to provide next level cell-to-network analysis by enabling the selective manipulation of subpopulations of cells and specific neuronal pathways. Evidence for retrosplenial involvement in the storage of long-term memory traces also comes from conditioning experiments where inactivation or re-activation of retrosplenial ensembles can abolish or reinstate retrieval, respectively [20,21^•^,^22^].

## Longitudinal imaging of rodent retrosplenial cortex

Inactivating RSC at different stages of task performance enables us to determine those processes for which RSC is critical. However, this approach is not without drawbacks; for example, a restricted number of inactivations can typically be assessed, there can be carry-over effects on subsequent learning and alterations to normal cell functioning over longer time periods. Critically, temporally altering cell firing in RSC may simply be telling us about the effects of interference across networks rather than how these networks function normally. Furthermore, this approach still potentially undervalues RSC involvement in tasks where RSC engagement is typical but not necessary. Measuring microstructural changes at different stages of learning can address some of these issues.

**Figure 2:**
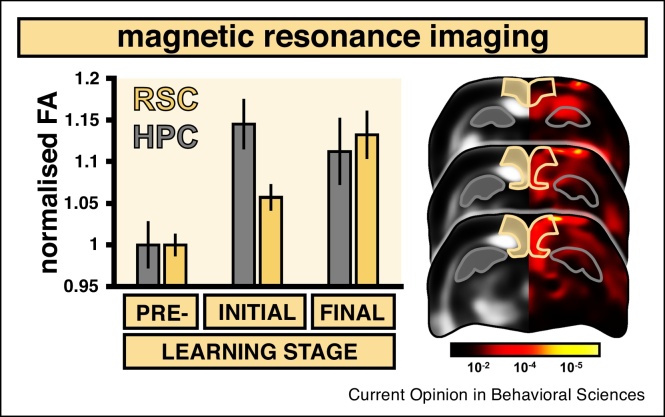
Diffusion tensor magnetic resonance imaging reveals learning-induced changes in grey matter microstructure. Training on a working-memory radial-arm maze task elicited differential patterns of fractional anisotropy (FA) changes in the RSC and the hippocampus (HPC), as displayed on the bar chart (error bars = S.E.M). While HPC FA values peaked at initial stages of learning, RSC signal saw a gradual increase with highest values upon mastering the task. The panel on the right displays a heatmap of *p*-values (calibration bar = Log_10_) from a repeated-measures ANOVA at three coronal brain levels showing significant differences in the HPC (in grey) and RSC (in yellow). Based on Figure adapted from Ref. [23].

Our group recently employed diffusion tensor imaging (DTI) to investigate changes in the microstructure of grey matter areas involved during spatial learning [23] (Figure 2). DTI measures tissue inhomogeneity resulting from the asymmetric movement of water molecules and changes to some of its metrics can capture plastic events in both humans and rodents [24, 25, 26]. In our study, animals were trained on a working memory version of the RAM task and we observed differential temporal engagement of the hippocampus and the RSC, with the former showing peak DTI changes during the initial stages of task acquisition. This contrasted with the RSC where the greatest changes were observed at the end of training when the animals were proficient at the task and the spatial environment had become familiar. This complementary engagement of the hippocampus and RSC is consistent with systems consolidation models where the hippocampus is engaged in rapid, early encoding and the cortex is involved in slower, long-term learning requiring the maturation of its representations [27,28]. Likewise, these findings highlight the preferential engagement of RSC in familiar rather than novel spatial environments, which is in line with human fMRI findings [29^••^].

## Increasing the resolution: from structure to cells

This longitudinal MR imaging approach is beneficial as it allows for brain-wide assessments and also provides more direct comparisons with human studies. An added benefit of rodent studies is that behaviour can be more closely controlled over longer time periods, enabling behaviour and neural changes to be more closely linked. However, MR imaging studies in rodents typically require animals to be anaesthetised which has a number of drawbacks, including limiting the overall number and frequency of scans that can be carried out. Furthermore, the spatial resolution of these scans makes it difficult to dissociate small subregions and be confident that changes are anatomically constrained.

An alternative is to use higher resolution methodology where individual cells can be assessed. IEGs, such as *c-fos*, *zif268* and *Arc*, are rapidly expressed in response to physiological or pharmacological stimulation and the proteins are subsequently involved in long-lasting adaptive changes [30]. As such, IEG imaging has been extensively used to probe the involvement of brain circuits in learning and memory and has increased our knowledge of RSC function. Initial experiments assessed IEG expression in post-mortem tissue. These studies revealed RSC engagement during the expression [31] and the consolidation of spatial memory task [32]; however, RSC is not differentially involved when performing the same task in a novel room or with a novel configuration of spatial cues, unlike hippocampus [31,33]. Again, this fits with the concept that RSC is not engaged in the processing of *novel* spatial cues [23,29^••^]. The resolution of IEG imaging enables analysis of subregions within RSC and this has provided additional evidence for functional dissociations within RSC: granular RSC is engaged in spatial working memory in both the light and the dark while dysgranular RSC is selectively involved in the light, that is, when visual cues are available [34]. These findings correspond with the dense connectivity between dysgranular RSC and visual cortex and the impaired use and integration of visual stimuli following selective dysgranular RSC lesions [5,6,35]. This IEG imaging approach has also been repeatedly used to demonstrate the sensitivity of RSC to diaschisis. There is a striking reduction in RSC activity, as measured by IEG imaging, following lesions of the hippocampus, anterior thalamic nuclei and the mammillothalamic tract [36, 37, 38, 39, 40], which likely contributes to the memory impairments associated with these lesions.

**Figure 3:**
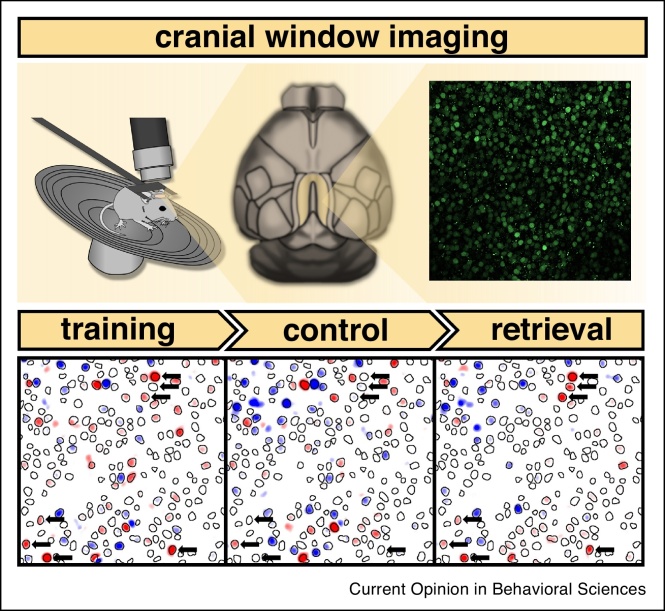
Longitudinal cranial window imaging of immediate-early gene expression in mice. Animals with windows implanted over the RSC (in yellow, seen from above, middle top panel) were repeatedly imaged to reveal the pattern of *c-fos* activity (top right panel — representative field of view) following acquisition and retrieval of spatial reference memory. Mice were head-fixed and placed on a running wheel under a two-photon microscope (top left) and z-stacks of the RSC were obtained. The bottom panel shows a representative pattern of active cells (in red) at the end of training, under a control condition and upon retrieval within the same field of view. Black outlines designate all identified cells while arrows indicate putative engram, that is memory trace, cells. Based on Figure adapted from Ref. [23].

This type of IEG imaging provides a method to look in-depth at multiple brain regions from the same animals. More recently, entire brains have been imaged [41], but with the limitation that only a single time-point can be assessed. A further development is using two-photon imaging to image cells longitudinally. The RSC is in many ways an ideal region for this approach given its location on the surface of the brain, simply requiring a window to be inserted in the overlying skull when imaging dysgranular RSC. Active neurons in the RSC, expressing an IEG, can be tagged with reporter genes, such as the green fluorescent protein, and repeatedly visualised under a cranial window [42]. We were able to use this approach to image the same cells over a number of days to examine the engagement of these cells during a spatial memory task [43] (Figure 3). Mice in the study were trained over a number of sessions on a reference memory task in the RAM. Over the course of training, a stable pattern of cell activity emerged, corresponding with the learning of the task. The pattern of neural activity was also re-instated upon retrieval of the task after a 24-day delay. Importantly, the fidelity of the re-instatement correlated with the animals’ performance levels, highlighting a role for the RSC in the encoding and storage of memory traces (engram formation). Furthermore, the time-scale of the developing engram is consistent with the findings from our DTI study, where RSC appears particularly important for the slow, long-term learning of spatial information. It also mirrors recent findings from Miller *et al.* [44^••^] who, using electrophysiological recordings, demonstrated the gradual involvement of the RSC throughout the acquisition of continuous spatial alternation in T-maze.

## From structure to networks

IEG analyses have enabled us to assess the impact of both distal lesions on RSC function and of RSC lesions on wider networks [40,45^•^]. While these studies highlight the importance of looking at the RSC in the context of wider networks, they fail to capture the dynamic nature of interactions across regions. A few studies have carried out simultaneous electrophysiological recordings of RSC and the hippocampus to examine the interplay between these structures. We and others have identified state-dependent effects on cross-frequency modulation within RSC and coherence between RSC and hippocampus [23,46]. The interactions between RSC and hippocampus during REM sleep may be particularly relevant for the RSC’s role in consolidation. Likewise, RSC-hippocampal coherence also varies with contextual fear learning such that the degree of RSC-hippocampal theta peak coherence can predict retrieval of remote fear memory [47]. Recent developments in probes and recording capacities will undoubtedly advance our understanding of RSC participation in large-scale memory networks.

## Novel techniques for exploring the temporal dynamics of memory

The use of direct functional imaging techniques as well as electrophysiological recordings offers a window into the temporal progression of mechanisms underlying memory formation, consolidation and retrieval. Imaging of genetically encoded calcium indicators expressed in select neuronal subpopulations yields critical information about both the identity and the activity patterns of mnemonic circuits. Consistent with the placement of the human RSC among scene selective areas, the rodent RSC shows sensitivity to both basic [48] and more complex visual stimuli providing contextual information. Calcium signals in RSC provide evidence for place-field like activity when traversing simple environments [49]. Such ‘place fields’ appear critically dependent on their hippocampal inputs and show gradual stabilisation over the course of learning [50^••^]. RSC also modulates visual cortex responses in mice that have learnt a visual avoidance task, showing RSC can exert top-down control over sensory responses and that control increases over training [51]. While very informative, calcium-imaging studies are still limited in their ability to replicate natural animal behaviours (e.g. due to head-fixation) and may be confounded by ectopic activity patterns [52]. Nevertheless, the use of microensdocopes in freely moving animals, including mesoscale level analyses (capturing large areas of the cortex simultaneously) [e.g., 53] may soon help overcome these shortcomings.

## Conclusions

No individual experimental approach can capture the complex nature of memory. While novel technological advancements have afforded unprecedented levels of analysis, thus allowing more mechanistic models of memory processes to emerge, it remains crucial not to overlook more traditional approaches (see Box 1). This is very much true for behavioural studies where appropriate designs and use of controls remain essential. After all, understanding memory requires the ability to link behaviour with internal processes while resisting the temptation to reduce it to a very limited set of laboratory tasks. For example, the increased focus on fear conditioning experiments in rodents may be difficult to reconcile with the RSC’s role in human memory processes [27]. Likewise, the focus on isolated processes or brain areas can prove misleading. The application of new-generation in-vivo electrophysiological probes or imaging of genetically encoded calcium indicators have already shown huge promise in disentangling the contributions of neural networks to memory while genetic manipulations now allow the selective alteration of memory traces. These techniques may perhaps one day enable the reinstatement of normal RSC function following diaschisis resulting from both lesions and dementia. This is clearly an important goal given the repeated findings, from numerous experimental approaches, and across species, of the importance of the RSC for spatial memory and for the long-term representation of spatial associations.

Box 1 **What happens when technological advances occur at a faster rate than our understanding of behaviour?**

• Over the last two decades there has been a rapid advance in technologies available to assess neuroscience processes without comparable advances in behavioural analyses. This has resulted in studies with state-of-the-art neuroscience techniques without accompanying well-designed behavioural assays.
• Rodents are often able to use multiple strategies to solve behavioural tasks and these can change depending on the stage of training; experimenters must be able to determine what cues are used and when to accurately interpret behaviour.
• Overreliance on stressful tasks (e.g., fear conditioning) for assessing memory processes in rodents may limit the extent to which we can extrapolate findings from animal studies to human episodic memory processes.
• To improve translatability in memory research we need to tap into common processes across species [e.g., 54], preferably using ethologically valid approaches. Spatial memory tasks can be particularly informative in this respect. But sensory, attentional and mnemonic aspects of tasks must be taken into consideration to properly interpret data obtained from these new neuroscience techniques [e.g., 43,55].

Alt-text: Box 1

## Conflict of interest statement

Nothing declared.

## References and recommended reading

Papers of particular interest, published within the period of review, have been highlighted as:

• of special interest
•• of outstanding interest

## CRediT authorship contribution statement

**Michal M Milczarek:** Conceptualization, Writing - original draft. **Seralynne D Vann:** Conceptualization, Funding acquisition, Writing - original draft, Writing - review & editing.
